# Glycosylation Pattern and *in vitro* Bioactivity of Reference Follitropin alfa and Biosimilars

**DOI:** 10.3389/fendo.2019.00503

**Published:** 2019-07-24

**Authors:** Laura Riccetti, Samantha Sperduti, Clara Lazzaretti, Danièle Klett, Francesco De Pascali, Elia Paradiso, Silvia Limoncella, Francesco Potì, Simonetta Tagliavini, Tommaso Trenti, Eugenio Galano, Angelo Palmese, Abhijeet Satwekar, Jessica Daolio, Alessia Nicoli, Maria Teresa Villani, Lorenzo Aguzzoli, Eric Reiter, Manuela Simoni, Livio Casarini

**Affiliations:** ^1^Unit of Endocrinology, Department of Biomedical, Metabolic and Neural Sciences, University of Modena and Reggio Emilia, Modena, Italy; ^2^International PhD School in Clinical and Experimental Medicine, University of Modena and Reggio Emilia, Modena, Italy; ^3^PRC, INRA, CNRS, IFCE, Université de Tours, Nouzilly, France; ^4^Unit of Neurosciences, Department of Medicine and Surgery, University of Parma, Parma, Italy; ^5^Department of Laboratory Medicine and Pathological Anatomy, Azienda USL, NOCSAE, Modena, Italy; ^6^Analytical Development Biotech Products, Merck Serono S.p.A. (an affiliate of Merck KGaA, Darmstadt, Germany), Rome, Italy; ^7^Azienda Unità Sanitaria Locale—IRCCS di Reggio Emilia, Department of Obstetrics and Gynaecology, Fertility Center, ASMN, Reggio Emilia, Italy; ^8^Center for Genomic Research, University of Modena and Reggio Emilia, Modena, Italy; ^9^Unit of Endocrinology, Department of Medical Specialties, Azienda Ospedaliero-Universitaria, Modena, Italy

**Keywords:** FSH, biosimilar, gonal-F, bemfola, ovaleap, glycosylation, assisted reproduction (ART)

## Abstract

Recombinant follicle-stimulating hormone (FSH) (follitropin alfa) and biosimilar preparations are available for clinical use. They have specific FSH activity and a unique glycosylation profile dependent on source cells. The aim of the study is to compare the originator (reference) follitropin alfa (Gonal-f®)- with biosimilar preparations (Bemfola® and Ovaleap®)-induced cellular responses *in vitro*. Gonadotropin N-glycosylation profiles were analyzed by ELISA lectin assay, revealing preparation specific-patterns of glycan species (Kruskal-Wallis test; *p* < 0.05, *n* = 6) and by glycotope mapping. Increasing concentrations of Gonal-f® or biosimilar (1 × 10^−3^-1 × 10^3^ ng/ml) were used for treating human primary granulosa lutein cells (hGLC) and FSH receptor (FSHR)-transfected HEK293 cells *in vitro*. Intracellular cAMP production, Ca^2+^ increase and β-arrestin 2 recruitment were evaluated by BRET, CREB, and ERK1/2 phosphorylation by Western blotting. 12-h gene expression, and 8- and 24-h progesterone and estradiol synthesis were measured by real-time PCR and immunoassay, respectively. We found preparation-specific glycosylation patterns by lectin assay (Kruskal-Wallis test; *p* < 0.001; *n* = 6), and similar cAMP production and β-arrestin 2 recruitment in FSHR-transfected HEK293 cells (cAMP EC_50_ range = 12 ± 0.9–24 ± 1.7 ng/ml; β-arrestin 2 EC_50_ range = 140 ± 14.1–313 ± 18.7 ng/ml; Kruskal-Wallis test; *p* ≥ 0.05; *n* = 4). Kinetics analysis revealed that intracellular Ca^2+^ increased upon cell treatment by 4 μg/ml Gonal-f®, while equal concentrations of biosimilars failed to induced a response (Kruskal-Wallis test; *p* < 0.05; *n* = 3). All preparations induced both 8 and 24 h-progesterone and estradiol synthesis in hGLC, while no different EC_50_s were demonstrated (Kruskal-Wallis test; *p* > 0.05; *n* = 5). Apart from preparation-specific intracellular Ca^2+^ increases achieved at supra-physiological hormone doses, all compounds induced similar intracellular responses and steroidogenesis, reflecting similar bioactivity, and overall structural homogeneity.

## Introduction

Follicle-stimulating hormone (FSH) is a heterodimeric glycoprotein hormone produced by the pituitary and acting on the gonads (1). In fertile women, FSH controls reproduction supporting ovarian granulosa cell proliferation and follicular growth by binding to its G protein-coupled receptor (FSHR) (2).

FSH shares a 92-amino acid residue α subunit with other glycoprotein hormones and has a 111-amino acid residue, hormone-specific β subunit (3). Two N-linked heterogeneous oligosaccharide populations are bound to each protein backbone subunit and are involved in hormone folding and half-life, receptor binding, and activation (4, 5). After gonadotropin binding, FSHR conformation rearrangements occur, triggering intracellular signal transduction. Gαs protein signaling leads to adenylyl cyclase stimulation and cyclic-AMP (cAMP)/protein kinase A (PKA)-pathway activation (6, 7), resulting in cAMP-response element binding protein (CREB) (8, 9) and extracellular-regulated kinase 1/2 (ERK1/2) (10) phosphorylation. These phospho-proteins are key players modulating steroidogenesis, proliferation and survival/apoptosis (8, 11), all molecular events underlying reproductive functions (12). Upon ligand binding, FSHR recruits other heterotrimeric Gα proteins, including Gαq and Gαi (13–16), as well as other interactors (17), linking FSH action to multiple intracellular signaling pathways, such as the rapidly-activated, phospholipase C-dependent (18), cytosolic calcium cation (Ca^2+^) release (19). FSHR internalization and recycling is mediated by β-arrestin 1 and 2, which triggers G protein-independent ERK1/2 signaling (20, 21).

FSH exists in a number of isoforms differing in content and composition of oligosaccharides attached to the protein backbone (22). FSH glycoforms were proposed as biased receptor ligands (5, 23, 24) due to isoform-specific contact with FSHR (25) and intracellular signaling (26). Glycosylation is a post-translational process influencing the isoelectric point (pI) and half-life of the gonadotropin (27). In women, more glycosylated and acidic FSH isoforms, mainly due to sialylation, exhibit a prolonged *in vivo* half-life due to reduced kidney clearance and are secreted mostly during the early and mid-follicular phase, compared to FSH basic glycoforms, which are predominant before ovulation (28, 29). Highly acidic FSH isoforms are produced more after the menopause than during the fertile lifespan (30), suggesting that glycoform composition of circulating hormones is dynamic and might have a physiological role.

Several formulations of exogenous FSH may be used in assisted reproductive technologies (ART) to induce multiple follicle development. Both urinary and recombinant FSH and other gonadotropin preparations are commercially available, as well as follitropin alfa biosimilar drugs, which are recombinant compounds similar to the originator (31–33). Previous studies attempted to address effects of these preparations on ART outcomes, given their different glycosylation states featured as post-translational modifications by the cellular source and/or purification processes (31, 34, 35). In fact, previous analyses by mass spectrometry found preparation-specific pattern of glycans bound to the FSH β-subunits (36, 37).

In this study, the biochemical composition and hormone-induced cell response of the originator follitropin alfa and two biosimilar preparations were analyzed *in vitro*. Glycosylation pattern was assessed in regard to cAMP production, Ca^2+^ release, β-arrestin 2 recruitment, CREB, and ERK1/2 phosphorylation and steroid (i.e., progesterone and estradiol) synthesis, which were analyzed in human primary granulosa-lutein cells (hGLC) and HEK293 cells transiently transfected with the human *FSHR* cDNA.

## Materials and Methods

### Follitropin Alfa Reference Preparation (Gonal-f®) and Biosimilars

The reference follitropin alfa and two biosimilar preparations were analyzed: Gonal-f® provided by Merck KGaA (Darmstadt, Germany), Ovaleap® purchased from Teva Pharmaceutical Industries (Tel Aviv, Israel) and Bemfola® from Finox Biotech (Kirchberg, Switzerland). Different batches of each preparation were tested by performing both biochemical and functional evaluations, as follows: two batches of Gonal-f® (AU016646, BA045956), two batches of Ovaleap® (S27266, R38915), and three batches of Bemfola® (PPS30400, PNS30388, PNS30230). Additional Gonal-f® (199F005, 199F049, 199F051) and Ovaleap® (S06622) batches were used for glycopeptide mapping. Comparison of hormone induced-signaling *in vitro* were performed by stimulating cells with gonadotropins concentrations expressed by mass rather than International Units (IU), since the latter depends of the *in vivo* activity in rats (38). Gonal-f® and biosimilar dosages were determined starting by the batch concentration declared by providers, consisting of 44 μg/ml for Gonal-f®, Ovaleap® and Bemfola®. Recombinant human choriogonadotropin (hCG; Ovitrelle®, Merck KGaA) was used as a negative control where indicated.

### Silver Staining and Western Blotting Analysis

According to gonadotropin quantification provided by the producers, 300 ng of each compound were subjected to 12% SDS-PAGE. Gel electrophoresis was performed under denaturing-reducing or *non-*denaturing*-non* reducing conditions, followed by silver staining and Western blotting. Denaturing conditions consisted of boiling samples 5 min at 100°C, while reducing conditions were obtained by adding 2-mercaptoethanol (Sigma-Aldrich, St. Louis, MO, USA), disrupting disulfide bonds (39). Silver staining was performed after acrylamide gel electrophoresis, as previously described (40, 41). Briefly, fixation was performed by incubating gels 1 h in 50% ethanol buffer, in the presence of 12% acetic acid and 5 × 10–4% formalin (all from Sigma-Aldrich). After washes, gels were stained with 0.2% AgNO_3_ buffer 30 min-treatment and signals were developed by 3% Na_2_CO_3_ buffer, 0.0005% formalin and 4 × 10–4% Na_2_S_2_O_3_ before to be stopped. Originator follitropin alpha and biosimilars were evaluated by Western blotting using a rabbit anti-human polyclonal primary antibody against FSHβ/FSH (SAB1304978; Sigma-Aldrich), while the secondary antibody was anti-rabbit human horseradish peroxidase (HRP)-conjugated (#NA9340V; GE HealthCare, Little Chalfont, UK). Recombinant hCG (Ovitrelle; Merck KGaA) was used as a negative control. Signals were developed with ECL (GE HealthCare) and acquired using the VersaDoc Imaging System (Bio-Rad Laboratories Inc., Hercules, CA, USA).

### Lectin ELISA Assay and Glycopeptide Mapping

The technique was described previously (41, 42) and adapted to preparations used in this study. A 96-well-microtiter plate was coated overnight at 4°C with the anti-human gonadotropin α subunit monoclonal antibody HT13.3 (43), which recognizes all human glycoprotein hormone α subunits, in 0.1 M sodium carbonate/hydrogen carbonate buffer (pH = 9.6). Plates were washed with a saline buffer (TBS-T; 25 mM Tris, 140 mM NaCl, 3 mM KCl, 0.05%, Tween 20; pH = 7.4) and *non*-specific sites were saturated by 1 h-treatment at room temperature (RT) using TBS-T containing 2% polyvinylpyrrolidone K30 (Fluka, Sigma-Aldrich). Duplicate 5 ng samples of each hormone preparation were then incubated over-night, in 100 μl/well of the saturation buffer. After washing, biotinylated lectins (Vector laboratories Ltd, AbCys Biologie, Paris, France) were placed into wells and incubated for 2 h at RT. Lectins used were: *Sambucus nigra* agglutinin (SNA), *Maackia amurensis* agglutinin (MAA), *Artocarpus Polyphemus* lectin (jacalin), *Ricinus communis* agglutinin (RCA-1, ricin), *Datura stramonium* agglutinin (DSA), wheat germ agglutinin (WGA), *Phaseolus vulgaris* agglutinin (PHA-E) (Supplemental Table 1). They were diluted in saturation buffer containing 1 mM CaCl_2_, 1 mM MgCl_2_, and 1 mM MnCl_2_. Plates were washed and peroxidase labeled NeutrAvidin^TM^ (Pierce, Interchim, Montluçon, France) was added in each well (100 μl in TBS-T), for 1 h at RT. After incubation with TMB ELISA peroxidase substrate standard solution (UP664781; Interchim, Montluçon, France) 20 min at RT, reactions were stopped by adding 50 μl/well of 2 N H_2_SO_4_, and absorbance measured at 450 nm wavelength using a spectrophotometer. Blank values, consisting of samples maintained in the absence of hormones, were subtracted to obtain ELISA data.

Additional information about reagents, glycopeptide mapping, hydrophilic interaction chromatography, and mass spectrometry analysis is provided in the supplemental section (Supplemental Material and Methods).

### Cell Culture and Transfection

HEK293 cells were cultured in Dulbecco's Modified Eagle Medium (DMEM) supplemented with 10% FBS, 4.5 g/l glucose, 100 IU/ml penicillin, 0.1 mg/ml streptomycin, and 1 mM glutamine (all from Sigma-Aldrich). Transient transfections were performed in 96-well plates using Metafectene PRO (Biontex Laboratories GmbH, München, Germany), in order to obtain exogenous FSHR and cAMP CAMYEL-, β-arrestin 2- or aequorin Ca^2+^-BRET biosensor protein expression (15), as previously described (41). For cAMP evaluation, 50 ng/well of FSHR-expressing plasmid were mixed together with 0.5 μl/well of Metafectene PRO in serum-free medium and incubated 20 min. A 50 μl aliquot of cAMP CAMYEL biosensor-expressing plasmid-Metafectene PRO mix was added to each well-containing 1 × 10^5^ cells, in a total volume of 200 μl/well, and incubated 2-days before stimulation with gonadotropins. One hundred ng/well of FSHR-Rluc8- and 100 ng/well of β-arrestin 2 biosensor-expressing plasmids were used for evaluating β-arrestin 2 recruitment. One hundred ng/well of FSHR- and 100 ng/well of aequorin biosensor-expressing plasmids were used to prepare cells for measure changes in intracellular Ca^2+^. All samples were prepared in duplicate and BRET measurements were performed using 2-day transfected cells, in 40 μl/well PBS and 1 mM Hepes.

Human primary granulosa lutein cells (hGLC) were isolated from ovarian follicles of about twenty donor women undergoing oocyte retrieval for ART, following written consent and with local Ethics Committee permission (Nr. 796 19th June 2014, Reggio Emilia, Italy). Patients had to match these criteria: absence of endocrine abnormalities and viral/bacterial infections, age between 25 and 45 years. Cells were recovered from the follicular washing fluid using a 50% Percoll density gradient (GE Healthcare, Little Chalfont, UK), following a protocol previously described (7, 44, 45). In order to restore expression of gonadotropin receptors (46), hGLC were cultured 6 days, then serum-starved over-night before use in experiments. Cells were cultured at 37°C and 5% CO_2_ in McCoy's 5A medium, supplemented with 10% FBS, 2 mM L-glutamine, 100 IU/ml penicillin, 100 μg/ml streptomycin and 250 ng/ml Fungizone (Sigma-Aldrich).

### BRET Measurement of cAMP Production, and β-arrestin Recruitment and Intracellular Ca^2+^ Increase

Intracellular cAMP and Ca^2+^ increase, and β-arrestin 2 recruitment were evaluated following a previously described procedure (15, 41, 47). Cyclic-AMP production and Ca^2+^ increase were evaluated in transiently transfected HEK293 cells using the *FSHR*-expressing plasmid, together with the BRET-based cAMP biosensor CAMYEL (48), or the aequorin Ca^2+^-biosensor expression vector (49), respectively, while BRET experiments cannot be performed in hGLC due to sub-optimal transfection efficiency and the high mortality rate in this cell model. Recruitment of β-arrestin 2 was assessed after transient transfection of HEK293 cells with the C-terminal, *Rluc*-tagged *FSHR* cDNA plasmid (provided by Dr. Aylin C. Hanyaloglu, Imperial College, London, UK) and N-terminal, yPET-tagged β-arrestin 2 (provided by Dr. Mark G. Scott, Cochin Institute, Paris, France). Cells were incubated 30 min in 40 μl/well PBS and 1 mM Hepes, in the presence or in the absence of increasing concentrations of Gonal-f® or biosimilars (1 × 10^−3^-1 × 10^3^ ng/ml range), and intracellular cAMP increase and β-arrestin 2 recruitment were measured upon addition of 10 μl/well of 5 μM Coelenterzine h (Interchim). A 4 × 10^3^ ng/ml hormone concentration-induced intracellular Ca^2+^ increase was evaluated over 100 s in transfected cells. Recombinant follitropin alfa or biosimilar addition occurred at the 25 s time-point. Light emissions were detected at 475 ± 30 and 530 ± 30 nm wavelengths using the CLARIOstar plate reader equipped with a monocromator (BMG Labtech, Ortenberg, Germany).

### Evaluation of ERK1/2 and CREB Phosphorylation

Hormone-induced ERK1/2 and CREB phosphorylation was analyzed by Western blotting following a protocol previously described (50). Human GLCs were seeded in 24-well plates (1 × 10^5^ cells/well) and treated for 15 min with increasing concentrations of gonadotropin (1 × 10^1^-1 × 10^3^ ng/ml range). Cells were immediately lysed for protein extraction in ice-cold RIPA buffer along with PhosStop phosphatase inhibitor and a protease inhibitor cocktail (Roche, Basel, Switzerland). Cell lysates were subjected to 12% SDS-PAGE and Western blotting, while pERK1/2 and pCREB activation were evaluated using specific rabbit antibodies (#9101 and #9198, respectively; Cell Signaling Technology Inc., Danvers, MA, USA). Sample loads were normalized to total ERK1/2 (#4695; Cell Signaling Technology Inc.). Membranes were treated with secondary anti-rabbit HRP-conjugated antibody (#NA9340V; GE HealthCare) and signals developed with ECL (GE HealthCare). Signal detection employed the VersaDoc system using the QuantityOne analysis software (Bio-Rad Laboratories Inc.). Protein density volumes were semi-quantitatively evaluated by the ImageJ software (U. S. National Institutes of Health, Bethesda, MD, USA) (51).

### Gene Expression Analysis

Hormone 50% effective concentrations (EC_50_s) were calculated from the cAMP dose-response curves and used for hGLC treatments before FSH-target gene expression analysis. Cells were seeded at 5 × 10^4^ cells/well in 24-well plates and exposed to gonadotropins for 8 h, and RNA was then extracted using the automated workstation EZ1 Advanced XL (Qiagen, Hilden, Germany). Equal amounts of total RNA were retrotranscribed by iScript reverse transcriptase (Bio-Rad Laboratories Inc.), according to a previously validated protocol (52). The expression of *STARD1* and *CYP19A1* genes encoding steroid-acute regulatory protein (StAR) and aromatase enzymes, respectively, was evaluated by real time PCR (7, 44) using specific primer sequences and protocols previously validated (7). Target gene expression was normalized to *ribosomal protein subunit 7* (*RPS7*) gene expression using the 2^−Δ*ΔCt*^ method (53). Experiments were recorded as the mean value of duplicates.

### Steroid Hormone Stimulation Protocol and Measurement

Human GLCs were seeded in 24-well plates (4 × 10^4^ cells/well) and treated 8 or 24 h with increasing hormone concentrations (1 × 10^−3^-1 × 10^3^ ng/ml). Where appropriate, 1 μM 4-androstene-3,17-dione (androstenedione; #A9630; Sigma-Aldrich) was added, as a substrate to be converted to estrogen by the aromatase enzyme. Stimulations were terminated by freezing samples and total progesterone or estradiol was measured in the cell media by an immunoassay analyzer (ARCHITECT second Generation system; Abbot Diagnostics, Chicago, IL, USA).

### Statistical Analysis

Data were graphically represented using box and whiskers plots, histograms, X-Y graphs and tables, and indicated as means ± standard error of means (SEM). Western blotting results were normalized to total ERK signals. Intracellular Ca^2+^ increase was represented as kinetics of acceptor emissions measured at 525 ± 30 nm, and area under the curve (AUC) values were extrapolated for comparisons between preparations. Dose-response curves for cAMP and β-arrestin 2 were obtained by data interpolation using *non*-linear regression. BRET data were represented as induced BRET changes by subtracting the ratio of donor/acceptor biosensor emissions of the untreated cells from the values of the stimulated cells. Data distributions were analyzed by D'Agostino and Pearson normality test, while differences were evaluated by Kruskal-Wallis or Friedman test with Dunn's multiple comparison *post-test* and considered significant when *p* < 0.05. Statistics were performed using the GraphPad Prism 6.01 software (GraphPad Software Inc., San Diego, CA, USA).

## Results

### Western Blotting and Silver Staining Analysis

**Figure 1:**
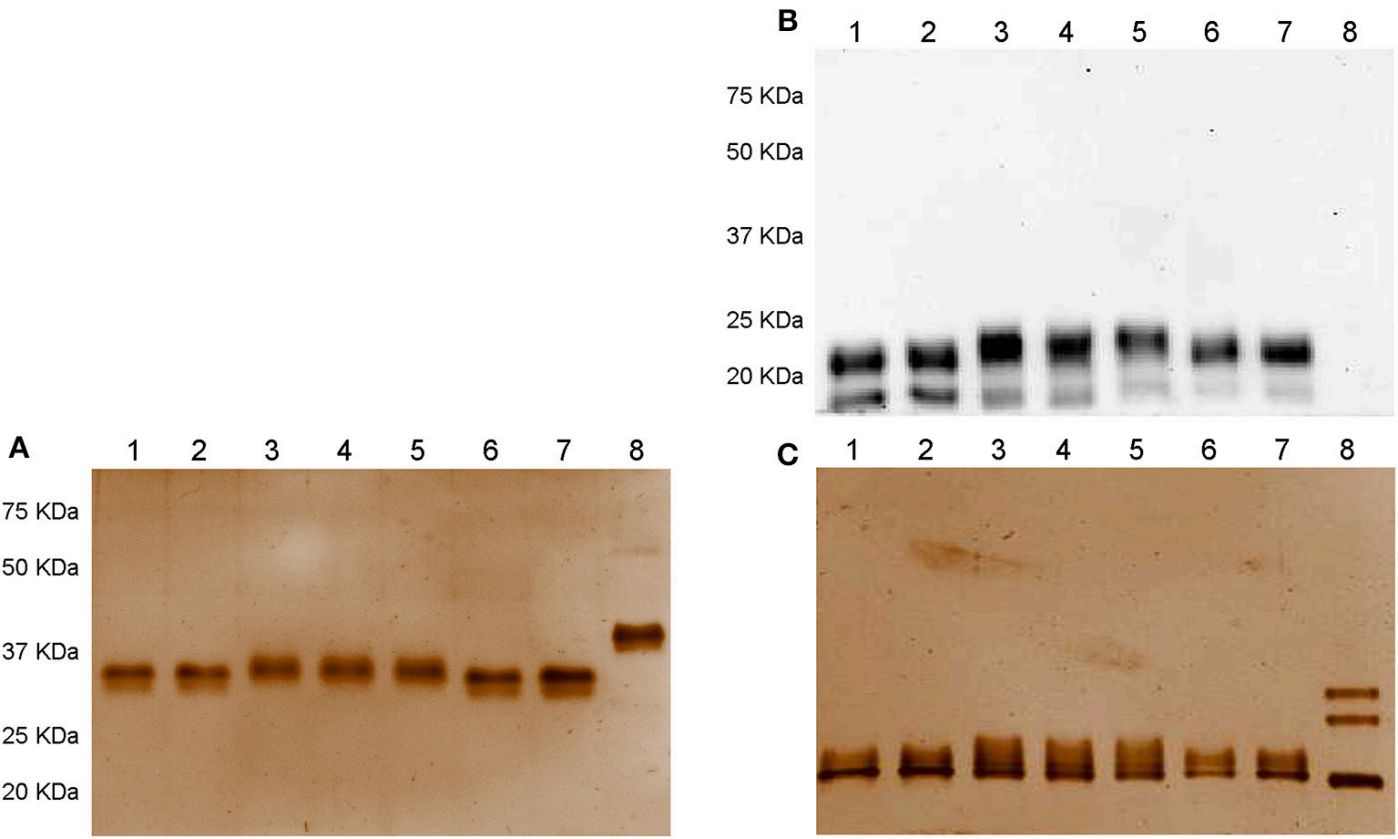
Western blotting **(A)** and silver staining analysis **(B,C)** of Gonal-f® and biosimilars under *non-*denaturing*-non* reducing and denaturing-reducing conditions. Samples comprising 300 ng of each preparation, according to the quantification provided by the manufacturer, were loaded. FSH presence was detected by rabbit anti-human polyclonal primary antibody against FSHβ/FSH. Recombinant hCG was used as negative control. Samples were loaded as follows: (1) Ovaleap® batch R38915, (2) Ovaleap® batch S27266, (3) Bemfola® batch PPS30400, (4) Bemfola® batch PNS30388, (5) Bemfola® batch PNS30230, (6) Gonal-f® batch AU016646, (7) Gonal-f® batch BA045956, (8) recombinant hCG. **(A)** Evaluation of FSH preparations under denaturing-reducing conditions, by Western blotting, using anti-FSHβ antibody. **(B)** Silver staining analysis of FSH preparations under *non-denaturing-non reducing* conditions. **(C)** Analysis of FSH preparations under denaturing-reducing conditions, by silver staining.

Samples comprising 300 ng/well of *non*-denatured and denatured Gonal-f® and biosimilar preparations were loaded onto a 12% acrylamide gel and separated by SDS gel electrophoresis under denaturing-reducing and *non-*denaturing*-non* reducing conditions. Denaturing conditions refer to 100°C-boiled samples, while reducing conditions were obtained by adding 2-mercaptoethanol. While no signals were detected under *non* denaturing-*non* reducing conditions by Western blotting (data not shown), two bands corresponding to the reference follitropin alfa and biosimilar preparations were revealed under denaturing-reducing conditions (Figure 1A). Ovaleap® and Gonal-f® preparations featured an ~20 KDa band, while a band corresponding to about 23 KDa molecular weight characterized Bemfola®. All preparations displayed a 15 KDa band of varying intensity. Recombinant hCG served as a negative control, providing no signal using the anti-FSH antibody.

Analysis by silver staining under *non* denaturing-*non* reducing conditions revealed that all preparations shared an overall similar protein pattern characterized by a single band at about 37 KDa molecular weight (Figure 1B). hCG resulted in a 40 KDa band. All samples displayed signals at about 20 KDa molecular weight (Figure 1C). Interestingly, no 15-KDa signals were detected, oppositely to that demonstrated by Western blotting, likely to be attributed to the low amount of FSHβ bound by the antibody and undetectable using silver staining due to sub-optimal sensitivity of this method (54). Three 35–20 KDa bands corresponding to recombinant hCG Ovitrelle® were detected, as previously described (41).

### Reference Follitropin Alfa and Biosimilar Reactivity to Lectins

The carbohydrate structure of follitropin alfa and biosimilars was investigated by ELISA, using a panel consisting of seven lectins characterized by specific recognition of different glycan features (Supplemental Table 1). Batches of each hormone were considered as experimental replicates and absorbance values measured at 450 nm were compared (Table 1).

**Table 1:** ELISA lectin analysis of reference and biosimilar follitropin alfa preparations.

**Lectins**	**Gonal–f^®^**	**Ovaleap®**	**Bemfola®**	***p*^a^**
	Absorbance (nm; means ± SEM*10^**3**^)	Absorbance (nm; means ± SEM*10^**3**^)	Absorbance (nm; means ± SEM*10^**3**^)	
MAA	74 ± 10	56 ± 1	60 ± 6	0.236
SNA	−2 ± 1	−35 ± 1	−40 ± 1	0.236
Jacalin	−1 ± 8	−9 ± 4	−17 ± 2	0.749
Ricin	120 ± 3	70 ± 2	180 ± 2	<0.0001
DSA	250 ± 8	370 ± 13	460 ± 8	0.001
PHA–E	1300 ± 30	1350 ± 40	1300 ± 30	0.814
WGA	100 ± 7	50 ± 5	80 ± 3	0.809

a *Kruskal Wallis test and Dunn's post-test*.

Bemfola® displayed structural peculiarities and variability, emerging by lectin analysis (Supplemental Table 2), due to significantly higher reactivity against ricin than other preparations (Kruskal-Wallis test; *p* < 0.05; *n* = 16; Table 1). Moreover, lectin assay revealed higher affinity of Bemfola® to DSA than Gonal-f® (Kruskal Wallis test; *p* < 0.05; *n* = 6). Ricin recognizes Galβ(1,4)GlcNAc monomers with higher affinity in the absence of sialylation in the terminal galactose, while DSA lectin binds Galβ(1,4) linked N-acetylglucosamine oligomers and a branched pentasaccharide sequence, including two N-acetyl lactosamine repeats linked to a mannose (55). No signal was detected with SNA lectin regardless of the hormone tested, indicating that sialic acid of the α(2,6) type is absent (56), likely due to the absence of galactoside α(2,6) sialyltransferase enzyme expression by CHO-K1 cells (57). Sialic acid of α(2,3) type is detected by MAA lectin in all samples (58), without any significant preparation-specific pattern. Jacalin failed to produce any signal, demonstrating the absence of O-glycans of the Galβ1-3GalNac or GalNac type (59). PHA-E lectin recognizes bi-antennary complex-type N-glycan with outer Gal and bisecting GlcNAc sequences (60), while WGA lectin reacts with GlcNAc sequences and sialic acid (61). Antennarity (Table 2), sialylation (Table 3), and sialic acid (Table 4) distribution were analyzed by glycopeptide mapping of Gonal-f® and Ovaleap® batches.

**Table 2:** Antennarity of reference and biosimilar follitropin alfa preparations.

**Glycosylation site**	**Antennarity distribution**	**Gonal-f^®^** **(means ± SEM)**	**Ovaleap^®^** **(means ± SEM)**	***p*^a^**
Asn52	Di-antennary	88.5 ± 0.5	90.6 ± 0.9	>0.999
	Tri-antennary	11.0 ± 0.6	9.1 ± 0.7	
	Tetra-antennary	0.4 ± 0.1	0.5 ± 0.2	
	A-Index	2.1 ± 0.0	2.1 ± 0.0	
Asn78	Di-antennary	91.5 ± 0.4	93.0 ± 0.5	>0.999
	Tri-antennary	8.3 ± 0.4	6.9 ± 0.2	
	Tetra-antennary	0.2 ± 0.1	0.2 ± 0.1	
	A-Index	2.1 ± 0.0	2.1 ± 0.0	
Asn7	Di-antennary	10.7 ± 0.4	6.0 ± 0.6	>0.999
	Tri-antennary	66.5 ± 1.1	73.2 ± 1.7	
	Tetra-antennary	19.3 ± 0.9	17.3 ± 1.7	
	One Repeat containing	3.3 ± 0.4	3.4 ± 0.5	
	A-Index	3.2 ± 0.0	3.2 ± 0.0	
Asn24	Mono-antennary	0.4 ± 0.1	0.3 ± 0.0	>0.999
	Di-antennary	87.5 ± 0.7	83.0 ± 1.2	
	Tri-antennary	7.7 ± 0.4	10.5 ± 0.3	
	Tetra-antennary	4.5 ± 0.3	6.1 ± 1.2	
	One Repeat containing	0.1 ± 0.0	0.3 ± 0.0	
	A-Index	2.2 ± 0.0	2.2 ± 0.0	

a *Kolmogorov-Smirnov test*.

**Table 3:** Sialylation distribution in reference and biosimilar follitropin alfa preparations.

**Glycosylation site**	**Sialylation indexes**	**Gonal-f^®^** **(means ± SEM)**	**Ovaleap^®^** **(means ± SEM)**	***p*^a^**
Asn52	S-extent (%)	96.0 ± 0.1	97.5 ± 0.2	>0.999
	S-index	2.0 ± 0.0	2.0 ± 0.0	
Asn78	S-extent (%)	85.0 ± 0.3	90.1 ± 0.2	0.400
	S-index	1.8 ± 0.0	1.9 ± 0.0	
Asn7	S-extent (%)	91.3 ± 0.2	95.4 ± 0.4	0.100
	S-index	2.9 ± 0.0	3.0 ± 0.0	
Asn24	S-extent (%)	88.0 ± 0.2	92.3 ± 0.7	0.100
	S-index	1.9 ± 0.0	2.0 ± 0.0	

a *Mann-Whitney's U-test*.

**Table 4:** Sialic acid distribution in reference and biosimilar follitropin alfa preparations.

**Glycosylation site**	**Sialic acid**	**Gonal-f^®^** **(means ± SEM)**	**Ovaleap^®^** **(means ± SEM)**	***p*^a^**
Asn52	NANA	97.3 ± 0.1	94.0 ± 0.1	>0.999
	NGNA	0.2 ± 0.1	4.2 ± 0.3	
	O-Acetylated NANA	2.5 ± 0.1	1.8 ± 0.3	
Asn78	NANA	95.0 ± 0.3	89.9 ± 0.2	>0.999
	NGNA	0.0 ± 0.0	4.4 ± 0.2	
	O-Acetylated NANA	5.0 ± 0.3	5.8 ± 0.4	
Asn7	NANA	97.5 ± 0.4	95.4 ± 0.5	>0.999
	NGNA	0.0 ± 0.0	2.9 ± 0.2	
	O-Acetylated NANA	2.5 ± 0.4	1.7 ± 0.6	
Asn24	NANA	92.8 ± 0.3	90.2 ± 0.5	>0.999
	NGNA	0.2 ± 0.1	3.6 ± 0.1	
	O-Acetylated NANA	7.0 ± 0.4	6.2 ± 0.4	

a *Kolmogorov-Smirnov test*.

These features were similarly represented among preparations (Kruskal-Wallis test; *p* ≥ 0.05; *n* = 6), as well as among batches (Supplemental Results), at least in Gonal-f® and Bemfola® (Chi-square test; *p* ≥ 0.05), which appeared to be homogeneous, overall (Supplemental Tables 3–5).

### Evaluation of Intracellular cAMP Increase and β-Arrestin 2 Recruitment

**Figure 2:**
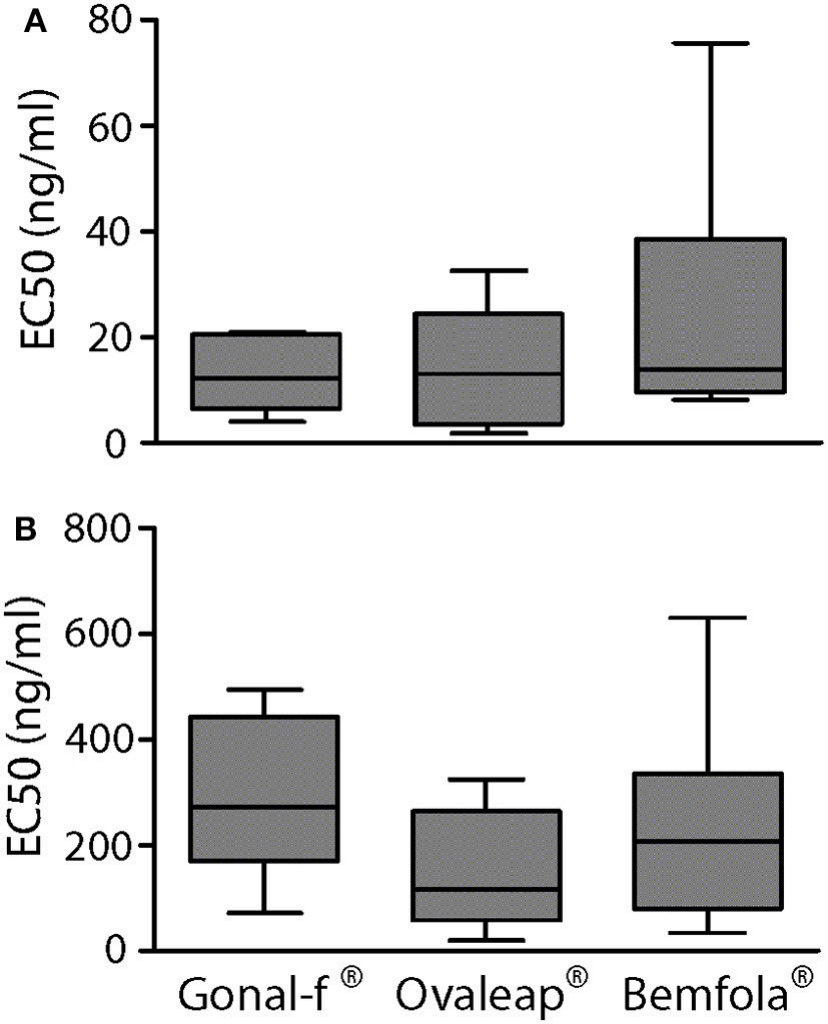
EC_50_ of cAMP response and β-arrestin 2 recruitment induced by Gonal-f® and biosimilars in transfected HEK293 cells. **(A)** Cells were transiently co-transfected with FSHR and CAMYEL sensor. cAMP was measured by BRET after 30 min stimulation with increasing doses of Gonal-f®, Ovaleap®, and Bemfola®. **(B)** Cells were transiently co-transfected with FSHR-Rluc8 and β-arrestin 2 –YPET sensors. β-arrestin 2 recruitment was measured by BRET after 30 min stimulation with increasing doses of hormones. EC_50_ values were extrapolated by non-linear regression. Data are represented as box and whiskers graphs (Kruskal Wallis test, *p* ≥ 0.05; *n* = 4).

Transfected, FSHR-expressing HEK293 cells were used to compare intracellular cAMP increases and β-arrestin 2 recruitment induced by 30-min treatment with increasing doses of reference follitropin alfa and biosimilars. Different batches of each preparation were tested and dose-response curves obtained by plotting cAMP and β-arrestin 2 levels in a semi-log X-Y graph (Supplemental Figure 1), in order to calculate and compare EC_50_ values obtained from the individual dose-response-curves (Figure 2).

Although preparation-specific carbohydrate structures were detected (Table 1), no significant differences were found between Gonal-f® and biosimilars' EC_50_ required for activating cAMP (Table 5; 12.9 ± 2.5–24.2 ± 6.0 ng/ml range; Kruskal-Wallis test, *p* ≥ 0.05; *n* = 4; Figure 2A) and β-arrestin 2 (Table 5; 140.7 ± 42.6–278.6 ± 56.9 ng/ml range; Kruskal-Wallis test, *p* ≥ 0.05; *n* = 4; Figure 2B), consistent between batches (cAMP: 10 ± 0.0–28 ± 0.0 ng/ml range; β-arrestin 2: 64 ± 0.0–610 ± 0.2 ng/ml range; Kruskal-Wallis test, *p* ≥ 0.05; *n* = 4; Supplemental Figure 1) and confirming similar potencies *in vitro*.

**Table 5:** Efficiency (EC_50_) of 30 min-cAMP and β-arrestin 2 production induced by reference and biosimilar follitropin alfa preparations in transfected, FSHR-expressing HEK293 cells.

**Preparation**	**EC_**50**_ cAMP** **(ng/ml; means ± SEM; *n* = 4)**	***p^a^***	**EC_**50**_ β-arrestin** **2 (ng/ml; means ± SEM; *n* = 4)**	***p*^a^**
Gonal-f®	12.9 ± 2.5	0.561	278.6 ± 56.9	0.223
Ovaleap®	14.7 ± 3.9		140.7 ± 42.6	
Bemfola®	24.2 ± 6.0		234.9 ± 57.2	

a *Kruskal-Wallis test*.

### Analysis of pERK1/2 and pCREB Activation

**Figure 3:**
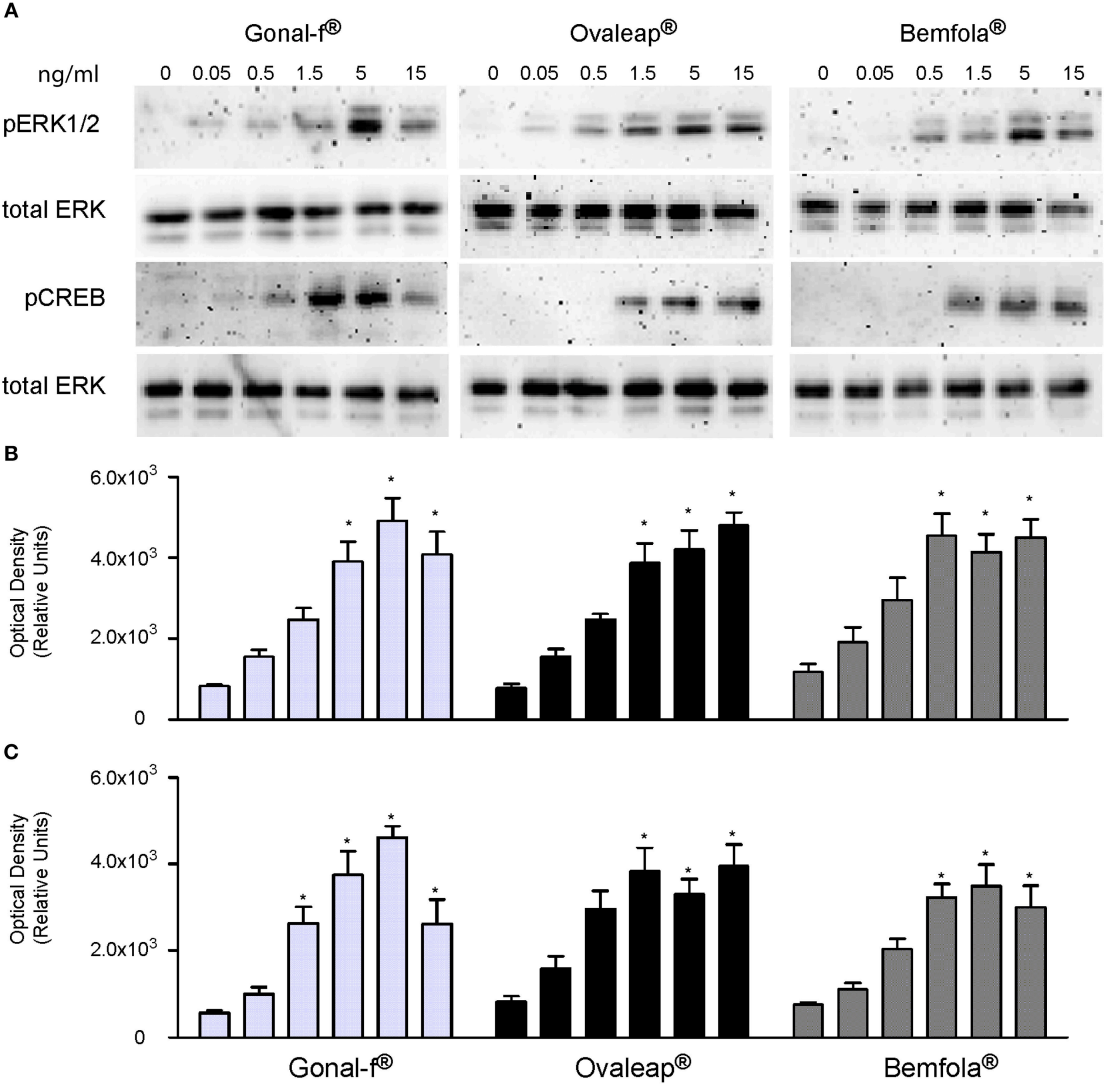
Evaluation of pERK1/2 and pCREB activation after Gonal-f® and biosimilars treatment of hGLC. Cells were stimulated by increasing doses of preparations. ERK1/2 and CREB phosphorylation were evaluated after 15 min by Western blotting (images representative of four independent experiments) **(A)**. **(B,C)** Densitometric analysis of pERK1/2 **(B)** and pCREB **(C)** signals. The values were normalized to total ERK and represented as means ± SEM, then statistically evaluated (* = significant vs. control (0 dose); Kruskal Wallis test; *p* < 0.05; *n* = 4).

The phosphorylation of ERK1/2 and CREB was evaluated in hGLC, which naturally express endogenous FSHR. Cells were treated for 15 min with increasing hormone concentrations, and phospho-protein activation was evaluated by Western blotting and semi-quantitatively measured (Figure 3). Total ERK served as a normalizer.

Similar ERK1/2 and CREB phosphorylation patterns were observed after stimulating cells with increasing doses of different batches of each preparation (Supplemental Figure 2). Mean results from batches of Gonal-f®, Ovaleap®, and Bemfola® were calculated and average hormone-specific pERK1/2 and pCREB activation results were reported (Figure 3). All preparations induced protein phosphorylation within the 1.5–15 ng/ml range (Kruskal-Wallis test, *p* < 0.05; *n* = 4), consistently between different batches of each preparation (Friedman test, *p* ≥ 0.05; *n* = 4). While no statistically significant differences between Gonal-f® and biosimilars' patterns of ERK1/2 phosphorylation were detected, pCREB activation occurred upon cell treatment by 0.5 ng/ml Gonal-f, differently to that obtained using both biosimilars (Friedman test, *p* < 0.05; *n* = 4). Interestingly, cell treatment by Gonal-f® and Bemfola® maximal concentrations (15 ng/ml) resulted in slightly decreased levels of CREB phosphorylation, not differing, however, significantly from the *plateau* levels of pCREB activation.

### *STARD1* and *CYP19A1* Gene Expression Analysis

**Figure 4:**
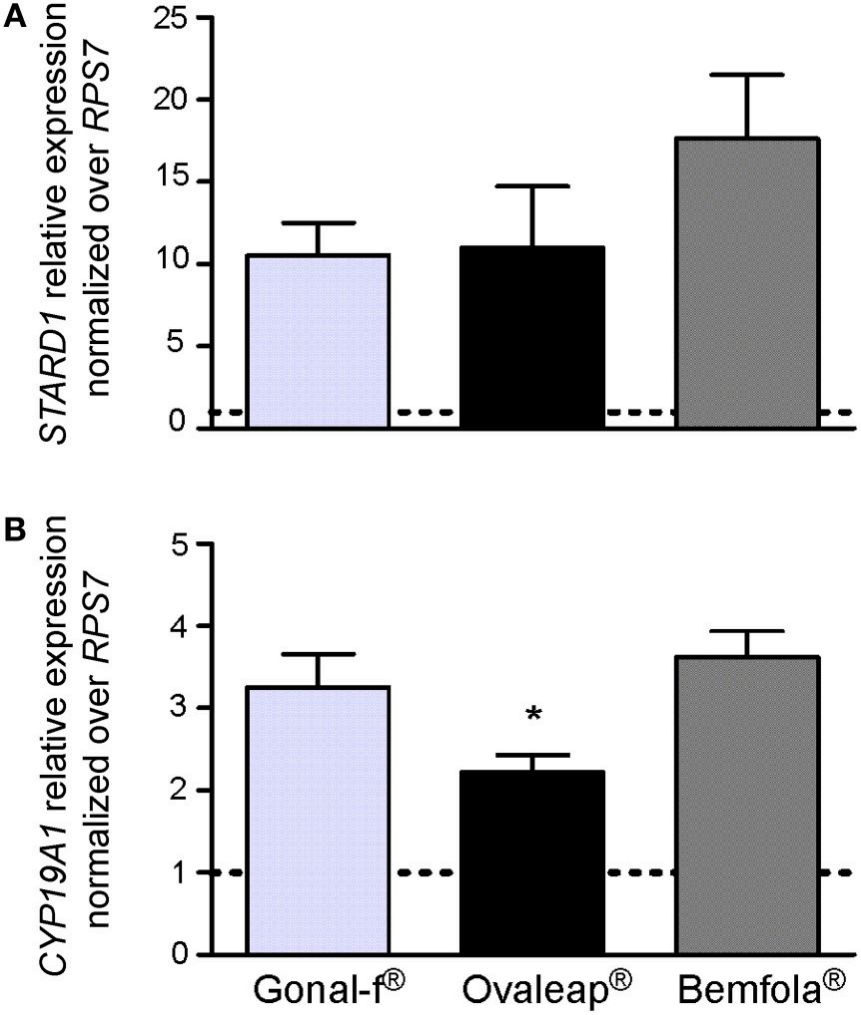
*STARD1* and *CYPA19A1* gene expression analysis. The expression of *STARD1* **(A)** and *CYPA19A1* **(B)** gene was evaluated in hGLC stimulated for 12 h with the EC_50_ of Gonal-f® or biosimilars (12 ng/ml Gonal-f® and Ovaleap®, 24 ng/ml Bemfola®) by real-time PCR. Each value was normalized over the *RPS7* gene expression (means ± SEM; *n* = 4). Unstimulated cells served as control and are indicated as a dotted line. (*= significant vs. Bemfola®, Kruskal Wallis test; *p* < 0.05).

Expression of FSH target genes was analyzed by real time PCR in hGLC. For this purpose, cells were stimulated 12 h by Gonal-f®, Ovaleap® or Bemfola®. Hormones were administered at the EC_50_ calculated from cAMP data (12 ng/ml Gonal-f® and Ovaleap®, 24 ng/ml Bemfola®). Total RNA was reverse-transcribed to cDNA and used for *STARD1* and *CYP19A1* gene expression analysis by real-time PCR. Data were normalized over the *RPS7* gene expression and represented as fold-increase over unstimulated cells in a bar-graph as means ± SEM (Figure 4).

Gonal-f®, Ovaleap®, and Bemfola® resulted in about 15-fold *STARD1* and 3-fold *CYP19A1* increase compared to the basal level (Kruskal-Wallis test, *p* < 0.05; *n* = 4). In particular, Ovaleap®-induced *CYP19A1* expression level lower than what was obtained by Bemfola® treatment (Kruskal-Wallis test, *p* < 0.05; *n* = 4). Treatment using different batches did not to affect *STARD1* and *CYP19A1* expression levels, since no significant differences between lots of any preparation occurred (Kruskal-Wallis test, *p* ≥ 0.05; *n* = 4; data not shown).

### Steroid Synthesis Analysis

Progesterone production and androgen-to-estrogen conversion were evaluated in hGLC treated for 8 or 24 h with hormones. For this purpose, cells were maintained under continuous stimulation by increasing gonadotropin concentrations (1 × 10^−3^-1 × 10^3^ ng/ml range) until reactions were stopped by freezing cell plates. To evaluate estradiol synthesis, androstenedione was added into wells as a substrate for the aromatase enzyme. Eight- and Twenty-four hours progesterone and estradiol dose-response curves were obtained and evaluated by *non*-linear regression, EC_50_ values calculated, and compared (Table 6).

**Table 6:** FSH EC_50_ values (ng/ml) in inducing 8 h- and 24 h-progesterone and estradiol production induced by reference and biosimilar follitropin alfa preparations (means±SEM; *n* = 5) in human primary granulosa cells.

**Preparation**	**Progesterone**	***p*^a^**	**Estradiol**	***p*^a^**
**8 h**				
Gonal-f®	1.5 ± 0.3		10.3 ± 4.4	
Ovaleap®	10.9 ± 3.7	0.285	5.6 ± 1.9	0.899
Bemfola®	4.4 ± 1.5		7.1 ± 2.6	
**24 h**				
Gonal-f®	15.4 ± 5.5		3.3 ± 1.0	
Ovaleap®	5.7 ± 1.2	0.799	2.5 ± 0.8	0.803
Bemfola®	7.3 ± 2.0		3.4 ± 1.0	

a *Kruskal-Wallis test*.

Reflecting cAMP accumulation, cell stimulation with Gonal-f®, Ovaleap®, and Bemfola® resulted in similar 8- and 24-h progesterone and estradiol production curves (Kruskal-Wallis test; *p* ≥ 0.05; *n* = 5), confirmed using different batches (Kruskal-Wallis test; *p* ≥ 0.05; *n* = 5; data not shown), as well as in similar progesterone and estradiol *plateau* levels (Kruskal-Wallis test; *p* ≥ 0.05; *n* = 5; Supplemental Table 6).

### Intracellular Ca^2+^ Increase

**Figure 5:**
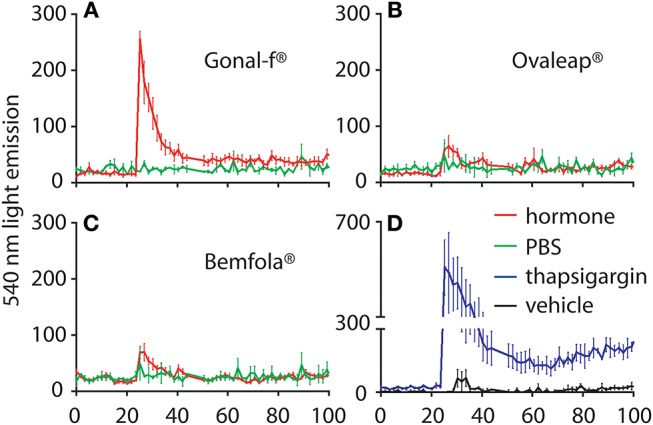
Calcium response kinetics in transfected HEK293 cells treated with Gonal-f® or biosimilars. Cells were transiently co-transfected with FSHR and aequorin sensors, then stimulated in duplicates with a fixed dose (4 × 10^3^ ng/ml) of **(A)** Gonal-f®, **(B)** Ovaleap®, **(C)** Bemfola®. **(D)** Thapsigargin, PBS and hormone diluent were used as positive and negative controls, respectively. BRET signal was measured for 100 s. Data are represented as means ± SEM (*n* = 3). Area under the curve (AUC) values were calculated and differences were considered for *p* < 0.05 (Kruskal Wallis test).

Kinetics of intracellular Ca^2+^ increase was evaluated in a transiently transfected HEK293 cell line that co-expressed both FSHR*-* and Ca^2+^-biosensors, by BRET. Cells were monitored for over 100 s and 4 × 10^3^ ng/ml hormone addition occurred at the 25 s time-point (Figure 5). A 10–20-fold supra-physiological FSH concentration was used, compared to FSH serum levels described in cycling women (62), due to the lack of an intracellular Ca^2+^ signal at lower hormone concentrations (data not shown). Thapsigargin and vehicle treatment were used as positive and negative controls, respectively. Data were represented as means ± SEM. AUC values were calculated to compare preparation-specific intracellular Ca^2+^ increase.

Addition of vehicle failed to induce any intracellular Ca^2+^ increase, confirming the lack of activity exerted by the solvent used for hormone dilution on calcium response. After confirming the absence of batch-specific results (Kruskal-Wallis test; *p* ≥ 0.05; *n* = 3; data not shown), cell treatment by Gonal-f® induced rapid intracellular Ca^2+^ increase, which was about 230-fold higher than vehicle (Kruskal-Wallis test; *p* < 0.05; *n* = 3) and occurred within 1–2 s after hormone addition. Bemfola® and Ovaleap® induced only a minimal, not significant intracellular Ca^2+^ increase (Kruskal-Wallis test; *p* ≥ 0.05; *n* = 3). Maximal levels of intracellular Ca^2+^ were achieved under thapsigargin treatment, which served as a positive control and induced an about 600-fold greater increase compared to the basal level (Kruskal-Wallis test; *p* < 0.05; *n* = 3).

## Discussion

We compared the biochemical profiles and hormone-induced cell responses of the reference follitropin alfa (Gonal-f®) and two biosimilars, Ovaleap® and Bemfola®, *in vitro*, revealing overall comparable hormone-induced intracellular signaling and steroidogenesis. Only the originator follitropin alfa induced hormone-specific pattern of CREB phosphorylation and, at supra-physiological concentrations (62), intracellular Ca^2+^ increase to transfected, FSHR-expressing cell lines.

Several gonadotropin formulations are commercially available, differing by source, purification process, and purity. Clinicians choose freely what preparation or combination of preparations will be administered to women undergoing ART (35). These preparations may differ in oligosaccharide content and number of branches attached to the protein backbone (63), depending on the glycosyltransferases equipment of the source cell. Gonal-f® is expressed by Chinese hamster ovary (CHO) cell lines (64), ensuring high bioactivity and batch-to-batch consistency (65, 66). Bemfola® is produced by a pre-adapted dihydrofolate reductase deficient CHO (CHO DHFR-) host cell line (67) and has demonstrated similar efficacy and safety *in vivo* as compared to the reference follitropin alfa, in a multi-center phase 3 study (68). Ovaleap® is also produced by a CHO-derived cell line after adaptation to serum free conditions (69) and has been demonstrated to be similar to follitropin alfa *in vivo* in a phase 3 clinical study (70).

Gonal-f® and Ovaleap® share two similar Western blotting patterns under denaturing and reducing conditions, likely due to specifically glycosylated FSH β-subunits (36, 37), while Bemfola® featured a ~23 instead of 20 KDa band according to its specific glycosylation pattern detected by mass spectrometry (36). Most of these signals were confirmed by silver staining, except for the absence of the 15 KDa band, likely due to sub-optimal sensitivity of the method (54). Analysis of native proteins contained in Gonal-f® and Ovaleap® batches, which were obtained by omitting treatment of samples by 100°C-heating and 2-mercaptoethanol reduction, revealed a single 37 KDa band consistent with the FSH heterodimer (71), while Bemfola® resulted in slightly higher apparent molecular weight. On the other hand, lectin assay revealed higher DSA signal in Bemfola® than Gonal-f®, likely due to different multiantennary complex structures on N-glycans demonstrated by glycopeptide mapping (36), and suggesting Bemfola®-specific glycosylation patterns. Lower ricin binding to Ovaleap® than to Bemfola® and Gonal-f® indicated a different content of Galβ1-4GlcNAc molecules (72).

Naturally occurring variations in carbohydrate structures were characterized during the follicular phase of the cycle (22) and might affect FSH bioactivity *in vivo* (73). Highly glycosylated FSH isoforms prevail at the early stages, while serum levels of less-acidic (sialylated) glycoforms increase at the mid-cycle until ovulation (29), suggesting a functional role of glycosylation and sialylated structures in modulating FSH bioactivity (26). However, crystallographic structures of FSH in complex with the receptor ectodomain suggested that carbohydrates are not located in the binding interface between the hormone and FSHR (74, 75), making unclear the physiological role of FSH sugar residues in hormone activity. In fact, analysis of signaling cascades revealed that cell treatment by Gonal-f® and biosimilars resulted in similar dose-response curves for both cAMP and β-arrestin 2, as well as ERK1/2 phosphorylation pattern. These results were replicated using different batches and are strengthened by similar ratios between EC_50_s observed for cAMP and β-arrestin 2 recruitment, confirming previously reported results obtained with follitropin alfa (15). On the other hand, the crystallographic structure of the human FSH bound to the extracellular binding domain of FSHR was obtained using partially deglycosylated hormone-receptor complexes (74). Therefore, it might be not fully descriptive of the role of sugar chains linked to the hormone in binding the receptor, providing a basis for explaining preparation-specific features, such as the higher potency of Gonal-f® in inducing CREB activation. These characteristics are likely linked to a relatively wide FSH EC_50_ range of progesterone response (Table 6; from 1.5 ± 0.3 to 10.9 ± 3.7 ng/ml), although not significantly different, presumably due to biased signaling (76, 77) of preparations.

Preparation-specific glycosylation patterns may be reflected by cellular response to supra-physiological doses of FSH *in vitro*. Biosimilar compounds induce barely detectable Ca^2+^ increases in FSHR-expressing HEK293 cells, which differed to that of Gonal-f® as previously reported using human pituitary FSH (78). FSHR is known to modulate intracellular Ca^2+^ increase *via* a molecular mechanism involving the phospholipase C (19). However, Gonal-f®-induced Ca^2+^ increase was obtained by hormone concentrations usually not achieved *in vivo* (62), while cAMP activation, and ERK1/2 CREB phosphorylation occurs at FSH doses achievable in serum, suggesting a supraphysiological shift from Gαs to Gαq protein-mediated activation of intracellular signaling cascades (8, 79). These data should be confirmed in other cell models, such as hGLC, since the pattern of intracellular signaling pathways is cell-specific and depends on the number and variety of GPCRs located at the cell surface (12, 79–81). Most importantly, preparation-specific activation of cAMP/β-arrestin 2 and intracellular Ca^2+^ increase indicated that these hormones might act as biased ligands under particular conditions, as well as the high sensitivity of the cAMP response detectable *in vitro*.

Confirming similar, FSH-induced *STARD1* expression in hGLC, no differences in 8 and 24 h-progesterone and estradiol production between hormones was found, despite their structural peculiarities and lower Ovaleap®-induced *CYP19A1* expression levels. Previous studies reported preparation-specific intracellular signaling resulting in similar long-term effects, measurable as 24-h steroid production (41, 45). These data are reminiscent of the earlier debate about recombinant and urinary FSH preparations, which provided similar pregnancy rate *per* fresh transfer (35, 68), as well as similar pharmacokinetic profiles (82). However, the matter is still debated. Different ART outcomes, depending on the use of Bemfola® vs. Gonal-f®, were postulated, possibly explained by different glycosylation, especially sialylation patterns between the two preparations and/or higher batch-to-batch variability (36) and estradiol production (82), observed with Bemfola®. Further *in vivo* investigations and extensive clinical experience are necessary to characterize the possible occurrence of biosimilar preparation-specific effects (31).

## Conclusions

Different glycosylation profiles are characteristic of the follitropin alfa and subsequent biosimilar preparations, likely due to the specific enzymatic equipment of the source cell lines. These molecular peculiarities do not result in major preparation-specific signals mediated at the intracellular level and steroid synthesis, which were found to be overall similar when follitropin alfa and biosimilars are used at concentrations resembling those obtained under physiological conditions. In light of the specific molecular features of these commercial compounds and of the slight differences demonstrated by the present study, and considering the relevance of their use for clinical purposes, the comparison between the reference follitropin alfa and biosimilar preparations merits further investigations in a variety of experimental settings.

## Data Availability

The raw data supporting the conclusions of this manuscript will be made available by the authors, without undue reservation, to any qualified researcher.

## Ethics Statement

Human primary granulosa lutein cells (hGLC) were isolated from ovarian follicles of donor women undergoing oocyte retrieval for ART, under written consent and local Ethics Committee permission (Nr. 796 19th june 2014, Reggio Emilia, Italy).

## Author Contributions

LR wrote a manuscript draft, performed experiments, and data analysis. SS, CL, DK, FD, EP, SL, ST, EG, AP, and AS contributed to experiments and edited the manuscript. FP and TT provided scientific and methodological assistance and edited the manuscript. JD, AN, MV, and LA provided assistance to experimental procedures and manuscript editing. ER and MS provided scientific support, data interpretation, and manuscript drafting. LC provided experiment management, data analysis and interpretation, and manuscript editing.

### Conflict of Interest Statement

EG, AP, and AS are Merck KGaA employees. The remaining authors declare that the research was conducted in the absence of any commercial or financial relationships that could be construed as a potential conflict of interest.

## Supplementary Material

Supplemental Figure 1 Cyclic-AMP response and β-arrestin 2 recruitment induced by different batches of Gonal-f® and biosimilars in trasfected HEK293 cells. **(A–C)** Cells were transiently co-transfected with FSHR and the CAMYEL sensor. Cyclic-AMP was measured by BRET after 30 min stimulation with increasing doses of **(A)** Gonal-f®, **(B)** Ovaleap®, and **(C)** Bemfola® batches. **(D–F)** Recruitment of β-arrestin 2 was measured in FSHR-Rluc8 and β-arrestin 2-YPET biosensor-expressing cells by BRET, after 30-min treatment of with increasing doses of **(D)** Gonal-f®, **(E)** Ovaleap®, and **(F)** Bemfola®. Data were represented as means ± SEM. No significant differences between EC_50_ values were found (Kruskal Wallis test, *p* ≥ 0.05; *n* = 4).

Supplemental Figure 2 Densitometric analysis of pERK1/2 and pCREB activation induced by different batches of Gonal-f® and biosimilars in hGLC. Cells were stimulated with increasing doses of FSH preparations and 15 min- ERK1/2 **(A)** and CREB **(B)** phosphorylation evaluated by semi-quantitative Western blotting. Values were normalized to total ERK and represented as means ± SEM. Differences between batches of each preparation was statistically evaluated (Kruskal Wallis test; *p* ≥ 0.05; *n* = 4).

Supplemental Table 1 Lectin specific binding sites.

Supplemental Table 2 ELISA lectin analysis of different batches of originator and biosimilar follitropin alfa. Lectins were used as follows: MAA (*n* = 20), SNA (*n* = 14), Jacalin (*n* = 6), ricin (*n* = 16), DSA (*n* = 6), PHA-E (*n* = 8), WGA (*n* = 8). Hormone reactivity to lectins was represented as absorbance measured at 450 nm (means ± SEM)^*^10^3^, after subtracting values obtained in the absence of gonadotropin. Data were analyzed by Kruskal-Wallis test, taking *p* < 0.05 as significant.

Supplemental Table 3 Antennarity distribution of Gonal-f® and Ovaleap® batches.

Supplemental Table 4 Sialylation distribution of follitropin alfa and Ovaleap® batches. Asn^7^, Asn^24^, Asn^52^, and Asn^78^ were analyzed in terms of percentage.

Supplemental Table 5 Sialic acids distribution of follitropin alfa originator and Ovaleap® batches.

Supplemental Table 6 Eight h- and twenty four hours-progesterone and estradiol *plateau* levels induced by Gonal-f® and biosimilar stimulation of human primary granulosa cells. Data are represented as means ± SEM (Kruskal-Wallis test, *p* ≥ 0.05; *n* = 5).

Supplemental Data Sheet 1 Glycopeptide mapping.

Supplemental Data Sheet 2 Supplemental Results.
