# Supplementary figures and images for: Glycosylation Pattern and in vitro Bioactivity of Reference Follitropin alfa and Biosimilars
Source: Front Endocrinol (Lausanne). 2019 Jul 24;10:503. doi: 10.3389/fendo.2019.00503 (PMC6667556; doi:10.3389/fendo.2019.00503)

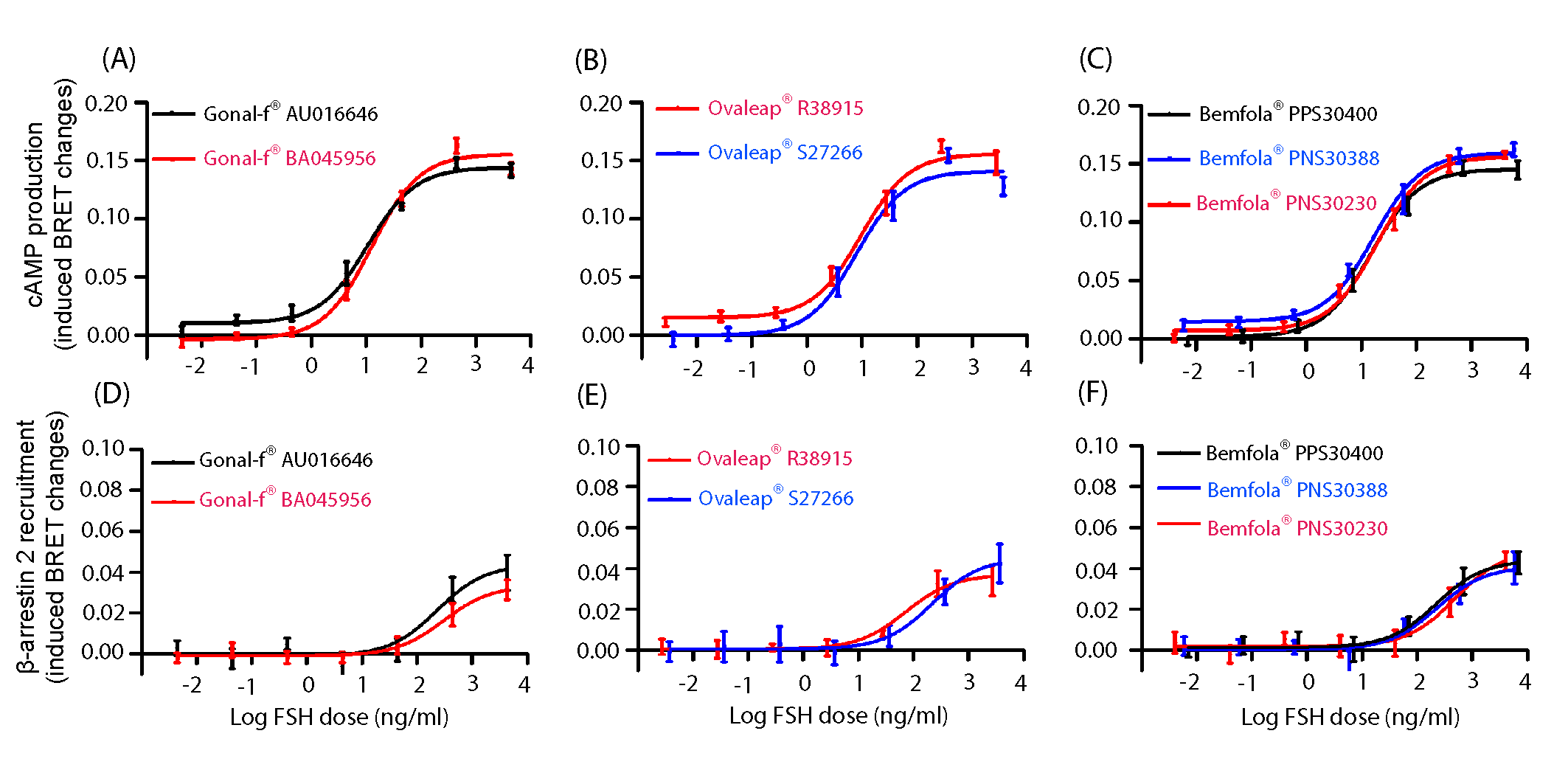

Supplement: Supplemental Figure 1 — Cyclic-AMP response and β-arrestin 2 recruitment induced by different batches of Gonal-f® and biosimilars in trasfected HEK293 cells. (A–C) Cells were transiently co-transfected with FSHR and the CAMYEL sensor. Cyclic-AMP was measured by BRET after 30 min stimulation with increasing doses of (A) Gonal-f®, (B) Ovaleap®, and (C) Bemfola® batches. (D–F) Recruitment of β-arrestin 2 was measured in FSHR-Rluc8 and β-arrestin 2-YPET biosensor-expressing cells by BRET, after 30-min treatment of with increasing doses of (D) Gonal-f®, (E) Ovaleap®, and (F) Bemfola®. Data were represented as means ± SEM. No significant differences between EC50 values were found (Kruskal Wallis test, p ≥ 0.05; n = 4). [file Image_1.TIF]

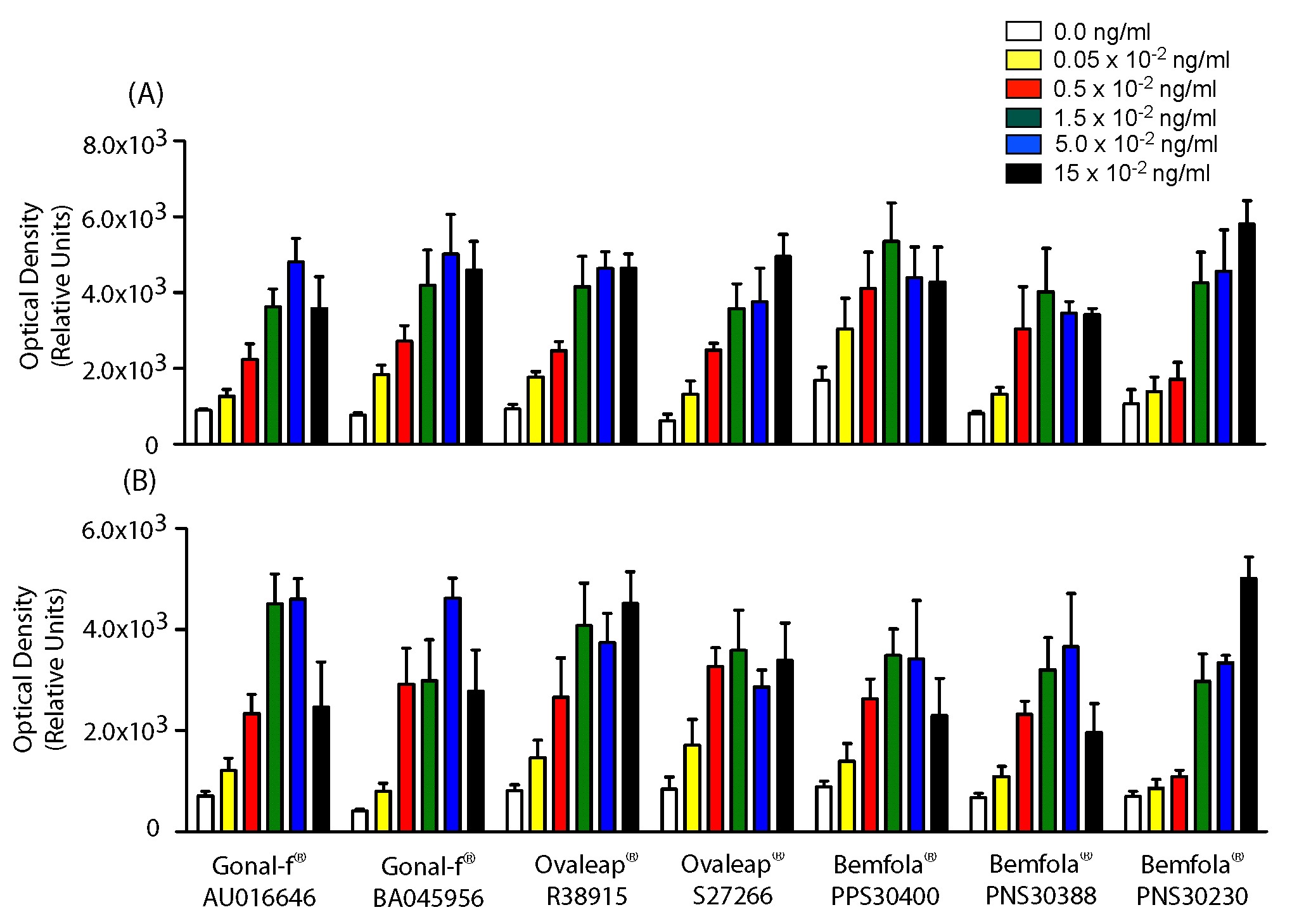

Supplement: Supplemental Figure 2 — Densitometric analysis of pERK1/2 and pCREB activation induced by different batches of Gonal-f® and biosimilars in hGLC. Cells were stimulated with increasing doses of FSH preparations and 15 min- ERK1/2 (A) and CREB (B) phosphorylation evaluated by semi-quantitative Western blotting. Values were normalized to total ERK and represented as means ± SEM. Differences between batches of each preparation was statistically evaluated (Kruskal Wallis test; p ≥ 0.05; n = 4). [file Image_2.TIF]
