# Supplementary material for: Glycosylation Pattern and in vitro Bioactivity of Reference Follitropin alfa and Biosimilars
Source: Front Endocrinol (Lausanne). 2019 Jul 24;10:503. doi: 10.3389/fendo.2019.00503 (PMC6667556; doi:10.3389/fendo.2019.00503)
Supplement: Supplemental Table 1 — Lectin specific binding sites. [file Table_1.docx]

**Supplemental Table 1. Lectins specific binding sites.**

| **Lectins** | **Name** | **Plant** | **Binding site** |
| --- | --- | --- | --- |
| MAA | *Maackia amurensis* agglutinin | *Maackia amurensis* | Neu5Acα2-3Gal |
| SNA | *Sambucus nigra* agglutinin | *Elderberry bark* | Neu5Acα2-6Gal(NAc) |
| Jacalin | jacalin | *Artocarpus integrifolia* | (Neu5Ac)Galβ1-3GalNAc (0-Glycans) |
| Ricin (RCA120) | *Ricinus communis* agglutinin 120 | *Ricinus communis* | Galβ1-4GlcNAc |
| DSA | *Datura stramonium* agglutinin | *Datura stramonium* | Pentasaccharide sequence including two  N-acetyllactosamine linked to a  mannose, (GlcNAcβ1-4) oligomers |
|  |  |  |  |
| PHA-E | *Phaseolus vulgaris* agglutinin | *Phaseolus vulgaris* | Bi-antennary complex-type N-glycan  with outer Gal and bisecting GlcNAc  sequences |
|  |  |  |  |
| WGA | wheat germ agglutinin | *Tritricum vulgaris* | GlcNAc, Neu5Ac |
