# Supplementary material for: Glycosylation Pattern and in vitro Bioactivity of Reference Follitropin alfa and Biosimilars
Source: Front Endocrinol (Lausanne). 2019 Jul 24;10:503. doi: 10.3389/fendo.2019.00503 (PMC6667556; doi:10.3389/fendo.2019.00503)
Supplement: Supplemental Table 2 — ELISA lectin analysis of different batches of originator and biosimilar follitropin alfa. Lectins were used as follows: MAA (n = 20), SNA (n = 14), Jacalin (n = 6), ricin (n = 16), DSA (n = 6), PHA-E (n = 8), WGA (n = 8). Hormone reactivity to lectins was represented as absorbance measured at 450 nm (means ± SEM)*103, after subtracting values obtained in the absence of gonadotropin. Data were analyzed by Kruskal-Wallis test, taking p < 0.05 as significant. [file Table_2.docx]

**Supplemental Table 2. ELISA lectin analysis of originator and biosimilar follitropin alfa batches.**

| **Preparation** Absorbance (nm) means±SEM x 10^3^ | **MAA** | p^a^ | **SNA** | p^a^ | **Jacalin** | p^a^ | **Ricin** | p^a^ | **DSA** | p^a^ | **PHA-E** | p^a^ | **WGA** | p^a^ |
| --- | --- | --- | --- | --- | --- | --- | --- | --- | --- | --- | --- | --- | --- | --- |
| Gonal-f^®^ AU016646 | 65±11 | 0.178 | 0.0±9 | 0.228 | 0.0±44 | 0.643 | 135±12 | 0.798 | 264±47 | 0.675 | 1240±160 | 0.494 | 92±37 | 0.950 |
| Gonal-f^®^ BA045956 | 85±11 |  | 7±12 |  | 0.0±26 |  | 125±27 |  | 228±31 |  | 1380±220 |  | 181±60 |  |
|  |  |  |  |  |  |  |  |  |  |  |  |  |  |  |
| Ovaleap^®^ R38915 | 60±9 | 0.673 | 0.0±10 | 0.236 | 0.0±22 | 0.853 | 85±15 | 0.308 | 360±61 | 0.788 | 1370±230 | 0.932 | 51±11 | 0.780 |
| Ovaleap^®^ S27266 | 52±7 |  | 0.0±6 |  | 0.0±15 |  | 53±12 |  | 382±76 |  | 1330±200 |  | 97±42 |  |
|  |  |  |  |  |  |  |  |  |  |  |  |  |  |  |
| Bemfola^®^ PPS30400 | 69±5 | 0.867 | 0.0±8 | 0.906 | 0.0±19 | 0.771 | 164±11 | <0.0001 | 494±71 | 0.670 | 1330±180 | 0.570 | 148±50 | 0.546 |
| Bemfola^®^ PNS30388 | 60±8 |  | 0.0±8 |  | 0.0±12 |  | 251±18 |  | 484±55 |  | 1290±190 |  | 111±35 |  |
| Bemfola^®^ PNS30230 | 62±7 |  | 0.0±7 |  | 0.0±17 |  | 137±13 |  | 414±57 |  | 1270±210 |  | 65±12 |  |

^a^ Kruskal Wallis test and Dunn's post-test
