# Supplementary material for: Glycosylation Pattern and in vitro Bioactivity of Reference Follitropin alfa and Biosimilars
Source: Front Endocrinol (Lausanne). 2019 Jul 24;10:503. doi: 10.3389/fendo.2019.00503 (PMC6667556; doi:10.3389/fendo.2019.00503)
Supplement: Supplemental Table 3 — Antennarity distribution of Gonal-f® and Ovaleap® batches. [file Table_3.docx]

**Supplemental Table 3. Antennarity distribution of Gonal-f**® **and Ovaleap**® **batches.**

| **Glycosylation site** | **Antennarity  distribution** | **Gonal-f^®^** | | |  | **Ovaleap^®^** | | |
| --- | --- | --- | --- | --- | --- | --- | --- | --- |
| **Batches** |  | 199F005 | 199F049 | 199F051 |  | S06622 | S27266 | R38915 |
| Asn52 | Di-antennary | 88.4 | 89.4 | 87.6 |  | 92.4 | 89.6 | 89.8 |
|  | Tri-antennary | 10.9 | 10.0 | 12.0 |  | 7.7 | 9.7 | 10.0 |
|  | Tetra-antennary | 0.5 | 0.5 | 0.3 |  | ND | 0.7 | 0.3 |
|  | A-Index | 2.1 | 2.1 | 2.1 |  | 2.1 | 2.1 | 2.1 |
|  |  |  |  |  |  |  |  |  |
| Asn78 | Di-antennary | 92.0 | 91.8 | 90.6 |  | 93.5 | 92.0 | 93.4 |
|  | Tri-antennary | 8.0 | 7.8 | 9.1 |  | 6.6 | 7.3 | 6.7 |
|  | Tetra-antennary | 0.1 | 0.1 | 0.3 |  | 0.1 | 0.4 | 0.2 |
|  | A-Index | 2.1 | 2.1 | 2.1 |  | 2.1 | 2.1 | 2.1 |
|  |  |  |  |  |  |  |  |  |
| Asn7 | Di-antennary | 11.3 | 10.9 | 9.8 |  | 6.6 | 4.9 | 6.6 |
|  | Tri-antennary | 64.8 | 68.5 | 66.3 |  | 74.6 | 69.9 | 75.2 |
|  | Tetra-antennary | 20.7 | 17.7 | 19.5 |  | 15.9 | 20.7 | 15.3 |
|  | One Repeat containing | 3.2 | 2.7 | 4.1 |  | 2.9 | 4.4 | 2.8 |
|  | A-Index | 3.2 | 3.1 | 3.2 |  | 3.2 | 3.2 | 3.1 |
|  |  |  |  |  |  |  |  |  |
| Asn24 | Mono-antennary | 0.3 | 0.3 | 0.7 |  | 0.2 | 0.3 | ND |
|  | Di-antennary | 89.0 | 86.8 | 86.8 |  | 83.5 | 84.9 | 80.7 |
|  | Tri-antennary | 6.9 | 7.8 | 8.3 |  | 10.0 | 10.5 | 10.9 |
|  | Tetra-antennary | 4.0 | 5.1 | 4.3 |  | 5.8 | 4.2 | 8.2 |
|  | One Repeat containing | 0.1 | ND | 0.1 |  | 0.2 | 0.3 | 0.3 |
|  | A-Index | 2.2 | 2.2 | 2.2 |  | 2.2 | 2.2 | 2.3 |
