# Supplementary material for: Glycosylation Pattern and in vitro Bioactivity of Reference Follitropin alfa and Biosimilars
Source: Front Endocrinol (Lausanne). 2019 Jul 24;10:503. doi: 10.3389/fendo.2019.00503 (PMC6667556; doi:10.3389/fendo.2019.00503)
Supplement: Supplemental Table 4 — Sialylation distribution of follitropin alfa and Ovaleap® batches. Asn7, Asn24, Asn52, and Asn78 were analyzed in terms of percentage. [file Table_4.docx]

**Supplemental Table 4. Sialylation distribution of Gonal-f^®^ and Ovaleap® batches.**

| **Glycosylation site** | **Sialylation  indexes** | **Gonal-f^®^** | | |  | **Ovaleap^®^** | | |
| --- | --- | --- | --- | --- | --- | --- | --- | --- |
| **Batches** |  | 199F005 | 199F049 | 199F051 |  | S06622 | S27266 | R38915 |
| Asn52 | S-extent (%) | 96.2 | 95.8 | 95.9 |  | 97.9 | 97.4 | 97.1 |
|  | S-index | 2.0 | 2.0 | 2.0 |  | 2.0 | 2.0 | 2.0 |
|  |  |  |  |  |  |  |  |  |
| Asn78 | S-extent (%) | 84.8 | 84.6 | 85.5 |  | 89.7 | 90.5 | 90.1 |
|  | S-index | 1.8 | 1.8 | 1.8 |  | 1.8 | 1.9 | 1.9 |
|  |  |  |  |  |  |  |  |  |
| Asn7 | S-extent (%) | 91.1 | 91.2 | 91.6 |  | 96.1 | 94.7 | 95.3 |
|  | S-index | 2.9 | 2.8 | 2.9 |  | 3.0 | 3.1 | 3.0 |
|  |  |  |  |  |  |  |  |  |
| Asn24 | S-extent (%) | 87.8 | 87.9 | 88.3 |  | 91.9 | 93.7 | 91.3 |
|  | S-index | 1.9 | 1.9 | 1.9 |  | 2.0 | 2.0 | 2.0 |
