# Supplementary material for: Glycosylation Pattern and in vitro Bioactivity of Reference Follitropin alfa and Biosimilars
Source: Front Endocrinol (Lausanne). 2019 Jul 24;10:503. doi: 10.3389/fendo.2019.00503 (PMC6667556; doi:10.3389/fendo.2019.00503)
Supplement: Supplemental Table 5 — Sialic acids distribution of follitropin alfa originator and Ovaleap® batches. [file Table_5.docx]

**Supplemental Table 5. Sialic acids distribution of Gonal-f^®^  and Ovaleap® batches.**

| **Glycosylation  site** | **Sialic acid** | **Gonal-f^®^** | | |  | **Ovaleap^®^** | | |
| --- | --- | --- | --- | --- | --- | --- | --- | --- |
| **Batches** |  | 199F005 | 199F049 | 199F051 |  | S06622 | S27266 | R38915 |
| Asn52 | NANA | 97.3 | 97.1 | 97.6 |  | 93.9 | 94.0 | 94.2 |
|  | NGNA | 0.3 | 0.3 | 0.0 |  | 3.9 | 4.0 | 4.7 |
|  | O-Acetylated NANA | 2.4 | 2.6 | 2.4 |  | 2.2 | 2.0 | 1.1 |
|  |  |  |  |  |  |  |  |  |
| Asn78 | NANA | 94.4 | 95.2 | 95.4 |  | 89.8 | 89.5 | 90.3 |
|  | NGNA | 0.0 | 0.0 | 0.0 |  | 4.6 | 4.0 | 4.5 |
|  | O-Acetylated NANA | 5.6 | 4.8 | 4.6 |  | 5.6 | 6.6 | 5.3 |
|  |  |  |  |  |  |  |  |  |
| Asn7 | NANA | 96.8 | 98.0 | 97.6 |  | 95.9 | 94.3 | 95.9 |
|  | NGNA | 0.0 | 0.0 | 0.0 |  | 3.3 | 2.7 | 2.6 |
|  | O-Acetylated NANA | 3.2 | 2.0 | 2.4 |  | 0.8 | 2.9 | 1.5 |
|  |  |  |  |  |  |  |  |  |
| Asn24 | NANA | 92.4 | 93.3 | 92.8 |  | 90.5 | 89.3 | 90.8 |
|  | NGNA | 0.0 | 0.4 | 0.1 |  | 3.5 | 3.7 | 3.6 |
|  | O-Acetylated NANA | 7.6 | 6.2 | 7.1 |  | 6.0 | 7.0 | 5.6 |
