# Supplementary material for: Glycosylation Pattern and in vitro Bioactivity of Reference Follitropin alfa and Biosimilars
Source: Front Endocrinol (Lausanne). 2019 Jul 24;10:503. doi: 10.3389/fendo.2019.00503 (PMC6667556; doi:10.3389/fendo.2019.00503)
Supplement: Supplemental Table 6 — Eight h- and twenty four hours-progesterone and estradiol plateau levels induced by Gonal-f® and biosimilar stimulation of human primary granulosa cells. Data are represented as means ± SEM (Kruskal-Wallis test, p ≥ 0.05; n = 5). [file Table_6.docx]

**Supplemental Table 6. Eight h- and 24 h-progesterone and estradiol *plateau* levels induced by reference and biosimilar follitropin alfa preparations in human primary granulosa cells.**

| **Preparation** | **Progesterone *plateau*** (ng/ml; means±SEM; n=5) | p^a^ | **Estradiol *plateau*** (pg/ml; means±SEM; n=5) | p^a^ |
| --- | --- | --- | --- | --- |
| **8 h** |  |  |  |  |
| Gonal-f® | 21.9±3.6 |  | 3052±2185 |  |
| Ovaleap® | 32.7±4.1 | 0.053 | 2090±413 | 0.945 |
| Bemfola® | 27.6±3.6 |  | 2576±597 |  |
| **24 h** |  |  |  |  |
| Gonal-f® | 29.3±5.6 |  | 5971±2687 |  |
| Ovaleap® | 51.6±8.6 | 0.260 | 4682±1190 | 0.674 |
| Bemfola® | 55.3±8.4 |  | 5202±1366 |  |
| ^a^ Kruskal-Wallis test | |  |  |  |
