# Supplementary material for: Glycosylation Pattern and in vitro Bioactivity of Reference Follitropin alfa and Biosimilars
Source: Front Endocrinol (Lausanne). 2019 Jul 24;10:503. doi: 10.3389/fendo.2019.00503 (PMC6667556; doi:10.3389/fendo.2019.00503)
Supplement: Supplemental Data Sheet 1 — Glycopeptide mapping. [file Data_Sheet_1.docx]

**Supplemental Material and Methods: glycopeptide mapping**

**Reagents**

Urea, iodoacetamide (IAM) and dithiothreitol (DTT) were purchased from Sigma (St. Louis, MO, USA). Chymotrypsin sequencing grade was purchased from Promega Corporation (Madison, WI, USA). Tris-(hydroxymethyl)-aminomethane (TRIS), ethylenediaminetetraacetic acid (EDTA), trifluoroacetic acid (TFA), hydrochloric acid (HCl) and liquid chromatography-mass spectrometry (LC-MS)-grade acetonitrile (ACN) were obtained from Merck KGaA (Darmstadt, Germany). 8 M guanidine solution was purchased from Thermo Fisher Scientific (San Jose, CA, USA). All water used in experiments was purified with a Milli-Q system from Merck Millipore (Milford, MA, USA). Amicon Ultracel 3k centrifugal filters were purchased from Merck Millipore (Tullagreen, Carrigtwohill, Ireland) and the Acquity UPLC BEH glycan column 1.7 μm, 2.1 x 150 mm was purchased from Waters (Milford, MA, USA).

**Glycopeptide mapping**

Individual batches of Gonal-f^®^ and Ovaleap^®^ were concentrated to 1 mg/ml using Amicon Ultracel 3k centrifugal filters. 200 μg of protein was then resuspended in 200 μl of denaturation buffer containing 8 M guanidine-HCl, 130 mM Tris-HCl and 1 mM EDTA at pH 7.6, and reduction was performed by adding 20 μl of 500 mM DTT and stirring for 60 minutes at 37°C. Samples were subsequently alkylated by adding 40 μl of 500 mM IAM and stirring in the dark for 30 minutes at room temperature. The buffer containing the reduced and alkylated samples was then replaced by digestion buffer containing 2 M urea and 50 mM Tris-HCl at pH 8.0 by using Amicon Ultracel 3k centrifugal filters. Proteins were then digested at 37°C for 4 h using chymotrypsin with an enzyme:substrate ratio of 1:20.

**Hydrophilic interaction chromatography and mass spectrometry analysis**

Chymotrypsin digested protein was analyzed using a Xevo G2-S QTof mass spectrometer (Waters) equipped with an Acquity I-Class UPLC system (Waters Corporation, Milford, MA, USA). Peptides were separated on an Acquity BEH glycan UPLC column (1.7μm, 2.1 x 150 mm) and eluted with a mixture of 0.1% TFA in water and 0.1% TFA in ACN. A 30–55% gradient of 0.1% TFA in water was used to separate over 60 minutes the glycopeptides. Mass spectrometry was performed using the MS^E^ function for dataset acquisition in the data independent mode. MS^E^ function uses an intelligent approach and acquires alternating scans with low and high collision energies to obtain precursor ion information, as well as collision induced dissociation (CID) fragmentation data. The instrument was operated with the following parameters: capillary voltage 3 kV, sampling cone 25 V, source temperature 100°C, desolvation temperature 250°C, cone gas flow 10 L/H, desolvation gas flow 800 L/H, and scan range 100–2500 m/z. The mass spectrometry data were processed using MassLynx 4.1 software (Waters). The identity of the glycopeptides was manually assigned, and the relative distribution of the glycan species was obtained by using BiopharmaLynx 1.3.4 software (Waters). N-glycan species distributions have been further elaborated by grouping species based on antennarity and sialylation.
