# Supplementary material for: Glycosylation Pattern and in vitro Bioactivity of Reference Follitropin alfa and Biosimilars
Source: Front Endocrinol (Lausanne). 2019 Jul 24;10:503. doi: 10.3389/fendo.2019.00503 (PMC6667556; doi:10.3389/fendo.2019.00503)
Supplement: Supplemental Data Sheet 2 — Supplemental Results. [file Data_Sheet_2.docx]

**Supplemental Results**

**FSH glycopeptide mapping**

The identification of site-specific glycopeptides distribution was obtained for three Gonal-f^®^ and three Ovaleap^®^ batches. Since the analysis of Bemfola^®^ glycosylation was recently conducted using this technical approach (Mastrangeli et al*.*, 2017), these batches were not included in the present investigation. N-glycan distribution at the four glycosylation sites, Asn^52^ and Asn^78^ for α-subunit and Asn^7^ and Asn^24^ for β-subunit, was consistent across batches of both Ovaleap^®^ and Gonal-f^®^, reflecting similar pattern of antennarity (Chi-square test; p≥0.05) (Supplementary Table 3). Analysis of sialylation at all these glycosylation sites demonstrated similar sialic acid content between Gonal-f^®^ and Ovaleap^®^ batches (Chi-square test; p≥0.05) (Supplementary Table 4). In particular, content of sialic N-acetylneuraminic (NANA), O-acetylated NANA and N-Glycolylneuraminic acid (NGNA) was observed to be qualitatively similar between preparations (Chi-square test; p≥0.05) (Supplementary Table 5).
